# Disparities in First Dose COVID-19 Vaccination Coverage among Children 5–11 Years of Age, United States

**DOI:** 10.3201/eid2805.220166

**Published:** 2022-05

**Authors:** Neil Chandra Murthy, Elizabeth Zell, Hannah E. Fast, Bhavini Patel Murthy, Lu Meng, Ryan Saelee, Tara Vogt, Kevin Chatham-Stephens, Christina Ottis, Lauren Shaw, Lynn Gibbs-Scharf, LaTreace Harris, Terence Chorba

**Affiliations:** Centers for Disease Control and Prevention, Atlanta, Georgia, USA (N.C. Murthy, H.E. Fast, B.P. Murthy, R. Saelee, T. Vogt, K. Chatham-Stephens, C. Ottis, L. Shaw, L. Gibbs-Scharf, L. Harris, T. Chorba);; CDC COVID-19 Response Team, Atlanta (N.C. Murthy, E. Zell, H.E. Fast, B.P. Murthy, L. Meng, R. Saelee, T. Vogt, K. Chatham-Stephens, C. Ottis, L. Shaw, L. Gibbs-Scharf, L. Harris, T. Chorba);; US Public Health Service Commissioned Corps, Rockville, Maryland, USA (N.C. Murthy, B.P. Murthy, K. Chatham-Stephens);; Stat-Epi Associates, Inc., Ponte Vedra Beach, Florida, USA (E. Zell);; General Dynamics Information Technology Inc., Falls Church, Virginia, USA (L. Meng)

**Keywords:** COVID-19, respiratory infections, severe acute respiratory syndrome coronavirus 2, SARS-CoV-2, SARS, coronavirus disease, zoonoses, viruses, coronavirus, vaccines, vaccination coverages, BNT162 vaccine, disparities, United States

## Abstract

We analyzed first-dose coronavirus disease vaccination coverage among US children 5–11 years of age during November–December 2021. Pediatric vaccination coverage varied widely by jurisdiction, age group, and race/ethnicity, and lagged behind vaccination coverage for adolescents aged 12–15 years during the first 2 months of vaccine rollout.

Although more common among adults, severe coronavirus disease (COVID-19) and hospitalization can occur in children. Among >8,300 hospitalized children 5–11 years of age, 1/3 required intensive care (*1*,*2*). Children can transmit severe acute respiratory syndrome coronavirus 2 to others, highlighting the need for pediatric COVID-19 vaccinations. On November 2, 2021, the US Centers for Disease Control and Prevention (CDC) recommended the use of the Pfizer-BioNTech COVID-19 vaccine (Pfizer Inc., https://www.pfizer.com) in children 5–11 years of age. We analyzed first-dose vaccination coverage among children 5–11 years of age and stratified coverage by age group, sex, race/ethnicity, and jurisdiction.

## The Study

We analyzed COVID-19 vaccine administration data among children 5–11 years of age in the United States during November 2–December 31, 2021. We collected data that were reported to CDC from jurisdictions, pharmacies, and federal entities through immunization information systems, the Vaccine Administration Management System, and direct data submission by January 21, 2022 (Appendix). We calculated daily and cumulative total numbers of children receiving the first dose of Pfizer-BioNTech COVID-19 vaccine. We calculated vaccination coverage by dividing the number of children who received the first vaccine dose by the total population of children in the corresponding age group living in the defined jurisdiction. We stratified vaccine coverage by jurisdiction, age group (5–6, 7–8, and 9–11 years), and sex. We obtained the population size for children 5–11 years of age from the US Census Bureau 2020 Population Estimates (*3*). Among 82.1% of children 5–11 years of age for whom race and ethnicity data were available, we calculated the percentage of children receiving their first COVID-19 vaccine dose by race/ethnicity and compared this with the racial and ethnic makeup of the US population 5–11 years of age.

We did not conduct tests for statistical significance because these data reflect US population and not population samples. We used SAS version 9.4 (SAS Institute, Inc., https://www.sas.com) to perform analyses. This study was reviewed by CDC and conducted consistent with applicable federal law and CDC policy.

**Figure 1:**
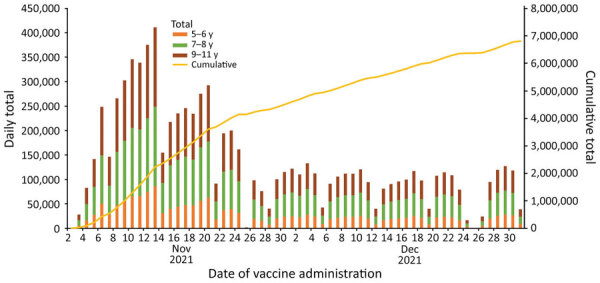
Daily and cumulative totals of the number of children 5–11 years of age who received the first dose of the Pfizer-BioNTech COVID-19 vaccine (Pfizer Inc., https://www.pfizer.com) by date of vaccination and age group, United States, November 2–December 31, 2021.

**Figure 2:**
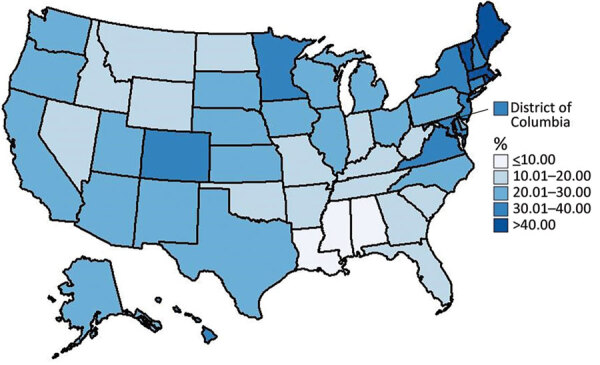
Percentage of vaccination coverage among children 5–11 years of age who received the first dose of the Pfizer-BioNTech COVID-19 vaccine (Pfizer Inc., https://www.pfizer.com), by jurisdiction, United States, November 2–December 31, 2021.

Overall, 24.0% of US children 5–11 years of age received their first COVID-19 vaccine dose during November–December 2021, and rapid initial uptake occurred during the first 2 weeks after CDC recommended the vaccine (Figure 1). Vaccination coverage varied by jurisdiction, ranging from 9.1% in Mississippi to 56.4% in Vermont. Coverage also varied by age group and was higher for children 9–11 years of age (26.8%) than children 5–6 years (20.3%) or 7–8 years (23.5%) (Figures 1, 2). Vaccination coverage did not vary by sex, 23.7% coverage for male children and 24.1% for female children (Appendix Table). 

Among all US children 5–11 years of age, non-Hispanic White persons constitute 51.2% of the population, non-Hispanic Black 14.0%, and Hispanic/Latino 23.0% (*3*). However, children from these groups were underrepresented among those reporting a first COVID-19 vaccination dose; only 49.1% non-Hispanic White, 8.0% non-Hispanic Black, and 21.7% Hispanic/Latino children were vaccinated. In contrast, among vaccine recipients, 11.4% were non-Hispanic Asian children, but this group constitutes only 5.6% of the US population 5–11 years of age (Appendix Figure).

## Conclusions

Vaccination coverage among children 5–11 years of age was only 24% and lagged vaccination coverage among children 12–15 years of age (33.3%) during the first 2 months of vaccine rollout (*4*). Many disparities among children 5–11 years of age emerged during the first 2 months of vaccine rollout, including racial and ethnic disparities. Children of Asian descent were overrepresented and White, Black, and Hispanic children were underrepresented. Many factors could explain these disparities. For instance, Asian Americans are less likely to live in poverty overall compared with other racial and ethnic groups (*5*). Poverty rates among Black (19.5%) and Hispanic (17.0%) communities are among the highest in the country (*6*), and lower income parents face challenges taking leave from work to get their children vaccinated or to care for children who have vaccine side effects (*7*).

Other factors that could hinder lower income parents from seeking vaccinations for their children include transportation challenges, a lack of pediatric and family medicine practices that serve as medical homes for routine pediatric care, and higher COVID-19 vaccine hesitancy among some parents (*8*,*9*). Access to a medical home could help address parental concerns about COVID-19 vaccines and improve vaccination uptake among pediatric populations. In addition, parental COVID-19 vaccination hesitancy varies by socioeconomic factors and is higher among parents whose children are publicly insured, such as through Medicaid, and parents in lower income social groups (*9*). Many factors influence parental hesitancy and additional concerted public health efforts to inform and educate parents and caregivers are needed to improve confidence in COVID-19 vaccines (*10*).

We found pediatric COVID-19 vaccination coverage varied widely across the United States and some jurisdictions had substantially higher vaccination coverage than others. Jurisdictions in the Northeast, including Vermont, Maine, Massachusetts, and Rhode Island, were among those with the highest vaccination coverage, and jurisdictions in the South, including Louisiana, Mississippi, and Alabama, were among those with the lowest coverage rates. This geographic variation could reflect parental vaccination status because adult vaccination coverage in the United States varied in a similar pattern (*4*). Parental COVID-19 vaccination status is one of the strongest predictors of pediatric COVID-19 vaccination (*11*), and efforts to build parental trust in COVID-19 vaccines are needed.

Furthermore, overall COVID-19 vaccination uptake among children 5–11 years of age was higher among children 9–11 years of age than children 5–6 years of age. The reasons for differences in vaccination coverage between the older and younger children are unknown but could reflect variations in parental hesitancy based on children’s ages. In a recent survey of parents of children 2–17 years of age, the younger the child, the less willing the parents were to vaccinate immediately (*11*). Among surveyed parents of children 5–11 years of age, 27% said they would get their children vaccinated for COVID-19 right away, but 33% said they would wait and see, 5% said they would only vaccinate if required, and 30% said they would definitely not get their children vaccinated (*7*).

CDC recommends that everyone >5 years of age receive COVID-19 vaccination to reduce illness and death (*12*). Pediatric and family medicine practices that serve as medical homes, along with pharmacies and other providers, should continue to promote and offer COVID-19 vaccines to children. Vaccination clinics hosted by schools, in collaboration with a vaccinating partner like a pharmacy or public health department, also might make vaccination convenient and help increase uptake of COVID-19 vaccination among children as they have done for routine vaccinations (*13*–*15*).

Our findings have >2 limitations. First, missing data on race and ethnicity for 17.9% of the records could bias findings by race/ethnicity, especially if differential reporting bias based on jurisdictions or by racial or ethnic subgroups occur. In addition, the US Census does not include a race category for “Other” as noted for many jurisdictions in immunization information systems. This finding could affect the interpretation of proportions for the “Multiple/Other, non-Hispanic” category because combining “Other” with “Multiple” in the immunization records could overrepresent vaccination coverage for this category. Finally, we calculated age for 14 jurisdictions where complete date of birth was unknown, which could have misclassified some age groups.

In conclusion, we found COVID-19 vaccination coverage among children 5–11 years of age varied substantially by jurisdiction, age group, and race or ethnicity. To ensure equity, jurisdictions nationwide should devise and implement strategic efforts to strengthen vaccination programs to build vaccine confidence and reduce barriers to receiving COVID-19 vaccines.

Appendix Additional information on emerging disparities in first dose COVID-19 vaccination coverage among children 5–11 years of age, United States.
