# Supplementary material for: Disparities in First Dose COVID-19 Vaccination Coverage among Children 5–11 Years of Age, United States
Source: Emerg Infect Dis. 2022 May;28(5):986–9. doi: 10.3201/eid2805.220166 (PMC9045440; doi:10.3201/eid2805.220166)
Supplement: Appendix — Additional information on emerging disparities in first dose COVID-19 vaccination coverage among children 5–11 years of age, United States. [file 22-0166-Techapp-s1.pdf]

Article DOI: https://doi.org/10.3201/eid2805.22016

Disparities in First-Dose COVID-19 Vaccination Coverage among Children 5–11 Years of Age, United States

Appendix

Data Sources

Immunization information systems are confidential, computerized, population-based systems that collect and consolidate vaccination data from providers in 64 public health jurisdictions and can be used to track administered vaccines and measure vaccination coverage. The 64 jurisdictions of these systems comprise the 50 US states, 8 US territories and freely associated states (Puerto Rico, US Virgin Islands, American Samoa, Commonwealth of the Northern Mariana Islands, Guam, Marshall Islands, Palau, and the Federated States of Micronesia), and 6 local jurisdictions (Chicago, Illinois; Houston and San Antonio, Texas; Philadelphia, Pennsylvania; New York City, New York; and Washington, DC).

The Centers for Disease Control and Prevention developed the Vaccine Administration Management System as a secure, online tool to manage vaccine administration from the time the vaccine arrives at a clinic until it is administered to a recipient (https://www.cdc.gov/vaccines/covid-19/reporting/vams/program-information.html). Direct data submission to the Centers for Disease Control and Prevention was available through the COVID-19 Vaccine Overview website (https://www.cdc.gov/vaccines/covid-19/reporting/overview/IT-systems.html).

Appendix Table. Vaccination coverage among children 5–11 years of age who received their first dose of the Pfizer-BioNTech COVID-19 vaccine, by jurisdiction, age group, and sex, United States, November 2–December 31, 2021*

	No. vaccinated (%)					
		Sex		Age group, y		
Jurisdiction	Total	F	M	5–6	7–8	9–11
United States	6,808,535 (24.0)	3,347,448 (24.1)	3,439,416 (23.7)	1,640,406 (20.3)	1,897,180 (23.5)	3,270,949 (26.8)
Alabama	39,688 (9.4)	19,782 (9.6)	19,886 (9.3)	8,747 (7.3)	10,776 (9.1)	20,165 (11.1)
Alaska	15,861 (22.2)	7,862 (22.5)	7,785 (21.3)	3,800 (18.4)	4,357 (21.1)	7,704 (25.4)
Arizona	144,286 (22.4)	71,224 (22.5)	72,968 (22.2)	34,460 (18.7)	40,485 (22.1)	69,341 (25.0)
Arkansas	42,701 (15.7)	20,510 (15.5)	20,784 (14.9)	9,639 (12.4)	11,880 (15.4)	21,182 (18.1)
California	962,980 (27.9)	477,893 (28.4)	484,187 (27.5)	221,394 (22.5)	266,092 (27.1)	475,494 (32.1)
Colorado	154,687 (31.7)	75,843 (31.9)	78,642 (31.6)	39,284 (28.6)	43,183 (31.5)	72,220 (33.9)
Connecticut	105,221 (38.3)	51,873 (38.5)	53,068 (37.9)	25,445 (33.3)	29,262 (37.8)	50,514 (41.7)
Delaware	19,046 (23.9)	9,493 (24.3)	9,540 (23.5)	4,684 (20.8)	5,210 (23.0)	9,152 (26.5)
District of Columbia	19,502 (37.3)	9,699 (37.5)	9,775 (37.0)	5,872 (36.0)	5,630 (35.9)	8,000 (39.5)
Florida	278,706 (16.9)	138,052 (17.1)	140,543 (16.7)	61,746 (13.1)	76,279 (16.2)	140,681 (19.8)
Georgia	140,352 (14.5)	69,593 (14.6)	70,374 (14.3)	32,320 (11.8)	38,792 (14.1)	69,240 (16.4)
Hawaii	39,104 (32.8)	19,002 (32.8)	20,054 (32.7)	8,535 (24.5)	10,751 (30.5)	19,818 (40.2)
Idaho	26,078 (14.7)	12,885 (14.9)	13,159 (14.6)	6,262 (12.6)	7,167 (14.4)	12,649 (16.4)
Illinois	319,100 (29.7)	156,511 (29.8)	162,354 (29.6)	76,586 (25.1)	88,965 (29.3)	153,549 (33.0)
Indiana	99,047 (16.3)	48,335 (16.3)	50,596 (16.2)	23,962 (13.8)	27,470 (15.8)	47,615 (18.2)
Iowa	62,142 (22.0)	30,527 (22.2)	31,487 (21.8)	15,613 (19.2)	17,554 (22.0)	28,975 (23.9)
Kansas	60,150 (22.0)	29,979 (22.4)	30,090 (21.5)	14,809 (19.2)	16,874 (21.7)	28,467 (24.0)
Kentucky	66,876 (17.2)	32,660 (17.3)	33,883 (17.0)	15,984 (14.3)	18,524 (16.7)	32,368 (19.5)
Louisiana	39,960 (9.5)	19,763 (9.6)	20,185 (9.4)	9,460 (7.7)	11,042 (9.3)	19,458 (10.8)
Maine	38,789 (40.7)	18,989 (40.9)	19,369 (39.7)	9,821 (36.6)	10,911 (40.5)	18,057 (43.5)
Maryland	181,843 (34.9)	89,720 (35.3)	91,853 (34.5)	45,664 (30.8)	50,837 (34.4)	85,342 (38.0)
Massachusetts	227,664 (44.1)	111,314 (44.1)	116,145 (44.1)	56,068 (38.5)	64,114 (43.6)	107,482 (48.2)
Michigan	185,024 (22.5)	90,830 (22.7)	94,155 (22.4)	46,432 (19.8)	51,464 (22.1)	87,128 (24.6)
Minnesota	163,829 (32.2)	79,866 (32.1)	82,685 (31.7)	41,374 (28.3)	46,727 (32.2)	75,728 (34.7)
Mississippi	24,165 (9.1)	12,064 (9.2)	12,080 (8.9)	5,159 (6.9)	6,391 (8.5)	12,615 (10.8)
Missouri	98,341 (18.5)	48,198 (18.6)	50,103 (18.4)	24,562 (16.2)	27,359 (18.1)	46,420 (20.3)
Montana	18,153 (20.0)	8,815 (20.0)	9,194 (19.7)	4,550 (17.4)	5,045 (19.5)	8,558 (22.0)
Nebraska	43,822 (23.5)	21,314 (23.6)	22,442 (23.4)	10,856 (20.1)	12,498 (23.7)	20,468 (25.8)
Nevada	40,480 (14.8)	20,031 (15.0)	20,446 (14.6)	8,816 (11.3)	11,123 (14.4)	20,541 (17.3)
New Hampshire	31,509 (32.5)	15,462 (32.7)	15,880 (31.9)	7,700 (28.7)	8,743 (31.8)	15,066 (35.2)
New Jersey	230,176 (30.8)	113,424 (31.0)	116,409 (30.5)	55,503 (26.3)	63,559 (29.9)	111,114 (34.3)
New Mexico	54,095 (29.1)	26,311 (28.8)	26,871 (28.4)	12,895 (24.9)	14,783 (28.1)	26,417 (32.3)
New York	485,354 (31.4)	236,785 (31.3)	245,536 (31.0)	118,709 (26.6)	133,969 (30.2)	232,676 (35.4)
North Carolina	199,406 (22.3)	97,743 (22.3)	100,459 (22.1)	47,202 (18.7)	55,448 (22.0)	96,756 (24.9)
North Dakota	12,845 (17.9)	6,236 (17.6)	6,532 (17.9)	3,148 (14.6)	3,719 (18.1)	5,978 (20.1)
Ohio	202,458 (20.3)	99,100 (20.4)	102,832 (20.2)	50,609 (17.7)	56,871 (20.0)	94,978 (22.3)

	No. vaccinated (%)					
		Sex		Age group, y		
Jurisdiction	Total	F	M	5–6	7–8	9–11
Oklahoma	51,704 (13.7)	25,537 (13.9)	26,158 (13.6)	12,206 (11.4)	14,303 (13.3)	25,195 (15.6)
Oregon	95,450 (28.1)	46,536 (28.1)	48,725 (27.9)	23,365 (24.2)	26,992 (28.0)	45,093 (30.6)
Pennsylvania	276,053 (27.1)	132,967 (26.7)	136,861 (26.3)	68,778 (23.8)	77,570 (26.7)	129,705 (29.6)
Rhode Island	33,754 (43.8)	16,818 (44.8)	16,885 (42.8)	8,295 (37.6)	9,388 (42.8)	16,071 (48.8)
South Carolina	60,960 (13.9)	30,292 (14.1)	30,599 (13.8)	13,799 (11.2)	16,729 (13.6)	30,432 (15.9)
South Dakota	18,936 (22.1)	9,294 (22.3)	9,428 (21.4)	4,591 (18.5)	5,291 (21.7)	9,054 (24.8)
Tennessee	77,516 (13.3)	38,200 (13.4)	39,259 (13.2)	18,636 (11.2)	21,595 (13.0)	37,285 (14.9)
Texas	606,580 (20.7)	299,641 (20.9)	306,712 (20.6)	146,478 (17.4)	170,448 (20.6)	289,654 (23.0)
Utah	96,237 (26.4)	47,333 (26.6)	48,815 (26.1)	23,797 (23.1)	27,090 (26.3)	45,350 (28.6)
Vermont	24,759 (56.4)	12,011 (57.0)	12,727 (55.8)	6,314 (51.0)	7,000 (56.1)	11,445 (60.2)
Virginia	241,733 (33.4)	118,593 (33.5)	122,992 (33.2)	57,781 (27.9)	68,150 (33.0)	115,802 (37.3)
Washington	197,138 (29.9)	96,757 (30.0)	99,850 (29.6)	50,665 (26.8)	55,679 (29.6)	90,794 (32.2)
West Virginia	20,661 (14.8)	10,124 (14.8)	10,257 (14.3)	4,536 (11.4)	5,755 (14.3)	10,370 (17.3)
Wisconsin	126,851 (26.0)	62,341 (26.2)	64,353 (25.8)	31,884 (23.2)	35,470 (25.7)	59,497 (28.0)
Wyoming	6,763 (12.9)	3,316 (13.0)	3,444 (12.7)	1,611 (11.0)	1,866 (12.6)	3,286 (14.2)

*Persons with state of residence reported as "unknown" (n = 10,591) were excluded from the table. Persons with sex reported as "unknown" (n = 21,671) were not represented in male and female counts (5–6 y, n = 6,642; 7–8 y, n = 5,748; 9–11 y, n = 9,281). COVID-19, coronavirus disease.

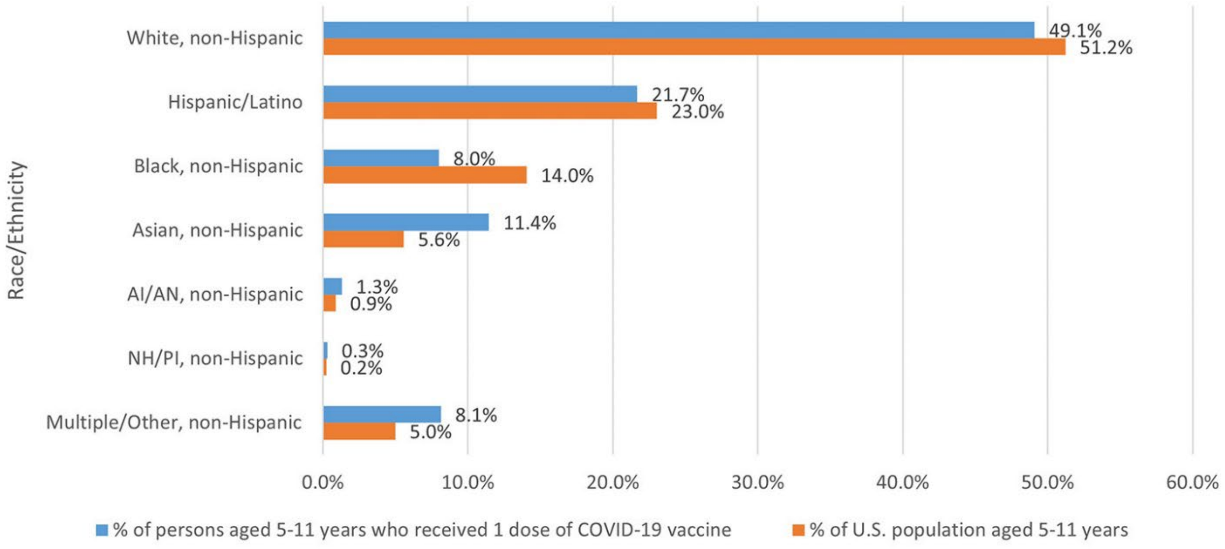

Appendix Figure. Race and ethnicity of children 5–11 years of age who received the first dose of the Pfizer-BioNTech COVID-19 vaccine (Pfizer Inc., https://www.pfizer.com), United States, November 2–December 31, 2021. Race and ethnicity information was available for 82.1% of persons included in the analysis. Of note, "Multiple/Other, non-Hispanic" represents persons identified as being non-Hispanic and having multiple race categories selected or being non-Hispanic and having "other" race selected. The US Census does not include "Other" as a race category, but many immunization information system jurisdictions might report "Other," which could affect the interpretation of proportions for this category. AI/AN, American Indian/Alaska Native; NH/PI, Native Hawaiian or Other Pacific Islander.
